# CamRegBase: a gene regulation database for the biofuel crop, *Camelina sativa*

**DOI:** 10.1093/database/baaa075

**Published:** 2020-12-11

**Authors:** Fabio Gomez-Cano, Lisa Carey, Kevin Lucas, Tatiana García Navarrete, Eric Mukundi, Steve Lundback, Danny Schnell, Erich Grotewold

**Affiliations:** Department of Biochemistry and Molecular Biology, 603 Wilson Road, Room 212, Biochemistry Building, East Lansing, MI 48824-6473, USA; Department of Plant Biology, Michigan State University, 612 Wilson Road, Room 166, East Lansing, MI 48824-1312, USA; Department of Biochemistry and Molecular Biology, 603 Wilson Road, Room 212, Biochemistry Building, East Lansing, MI 48824-6473, USA; Department of Biochemistry and Molecular Biology, 603 Wilson Road, Room 212, Biochemistry Building, East Lansing, MI 48824-6473, USA; Department of Biochemistry and Molecular Biology, 603 Wilson Road, Room 212, Biochemistry Building, East Lansing, MI 48824-6473, USA; Department of Biochemistry and Molecular Biology, 603 Wilson Road, Room 212, Biochemistry Building, East Lansing, MI 48824-6473, USA; Department of Plant Biology, Michigan State University, 612 Wilson Road, Room 166, East Lansing, MI 48824-1312, USA; Department of Biochemistry and Molecular Biology, 603 Wilson Road, Room 212, Biochemistry Building, East Lansing, MI 48824-6473, USA

## Abstract

Camelina is an annual oilseed plant from the Brassicaceae family that is gaining momentum as a biofuel winter cover crop. However, a significant limitation in further enhancing its utility as a producer of oils that can be used as biofuels, jet fuels or bio-based products is the absence of a repository for all the gene expression and regulatory information that is being rapidly generated by the community. Here, we provide CamRegBase (https://camregbase.org/) as a one-stop resource to access Camelina information on gene expression and co-expression, transcription factors, lipid associated genes and genome-wide orthologs in the close-relative reference plant *Arabidopsis*. We envision this as a resource of curated information for users, as well as a repository of new gene regulation information.

## Introduction

*Camelina sativa* is an emerging biofuel crop (1, 2). With a low economic input requirement (1), early season growth habit (3, 4), genetic similarity to the model plant *Arabidopsis* (5) and relatively high oil composition in the seed (6, 7), it has gained traction as a potential target for jet fuel and biodiesel production. Camelina’s genome has been sequenced and annotated, has a hexaploid genome structure harboring 89 418 protein-coding genes organized in 20 chromosomes (5, 8) and is relatively easy to genetically transform (9).

A challenge, albeit not unique to Camelina, is how to best utilize the burgeoning genomic information for predictive metabolic engineering of seed oil production (10, 11). Clearly, knowing how much and where gene expression takes place is necessary, as recently demonstrated by recent studies aimed at increasing oil production in Camelina using the co-expression of select genes (12). While RNA-Seq is a very powerful tool to determine global levels of gene expression, each analysis yields a large amount of data and therefore is non-trivial to curate and analyze for potential targets. In order to take advantage of all the currently available RNA-Seq data for Camelina, a relational database is the most ideal resource. Currently, Camelina genomics resources are part of the Brassica database BRAD (http://brassicadb.org) together with 11 Brassicaceae genomes. BRAD has a comparative approach to make plots of syntenic genomic regions and search the orthologs genes (13), but the most information available in BRAND is directed to *Brassica rapa*. In particular, the Camelina Genome Portal (camelinagenomics.org) allows a user to browse the whole Camelina genome assembly, conduct BLAST analyses to the Camelina genome, and view any of 15 946 (current number at date of publication) contig scaffolds on the sequenced genome. The University of Toronto has developed an electronic fluorescent pictograph browser (http://bar.utoronto.ca/) for *Camelina sativa*, which allows quick visual representation of expression data from a large developmental set. The Camelina Genomic Resources (camelinagenome.org) contains transcript data on protein and lipids but is only restricted to the developing embryo. Many databases also exist that provide information on TFs for one or multiple plants (14–21). AGRIS (https://agris-knowledgebase.org/), for example, provides a useful resource for the knowledgebase described here, because it provides a comprehensive collection of Arabidopsis TFs and other regulatory components, that can be easily translated to Camelina on the basis of the close relationship between these plants.

Here, we introduce the Camelina Gene Regulation Database (https://camregbase.org/), which is intended as a one-stop resource for aspects related to Camelina gene regulation. CamRegBase v1.0 harbors all RNA-Seq experiments available to-date with read abundance and the corresponding metadata, tissue-specific gene expression visualization and gene co-expression analyses. Additionally, CamRegBase 1.0 offers information on the orthologous relationships between Camelina and Arabidopsis genes along with the reported syntelog data (21). Finally, as a valuable resource to researchers interested in studying the control of gene regulation, CamRegBase 1.0 provides a comprehensive catalog of transcription factors (TFs) and co-activators identified by our own analyses and those previously reported (21) (http://planttfdb.cbi.pku.edu.cn/). With all the above-mentioned information integrated as one resource, CamRegBase is poised to become a primary resource for Camelina gene expression analyses.

## Materials and methods

### Gene expression data sources

Expression data present in CamRegBase 1.0 was retrieved from the Gene Expression Omnibus. All samples collected corresponded to RNA-Seq experiments generated using the Illumina platform. RNA-Seq results for a total of 131 experiments (including replicates) were collected, 40 of which corresponded to single-end libraries and 91 to paired-end libraries. These 131 experiments corresponded to a total of 16 different projects (Supplemental Table 1). All samples were subject to quality control using FastQC (http://www.bioinformatics.babraham.ac.uk/projects/fastqc/, V0.11.5). Libraries with adapters and reads with low quality (Phred < 20) were removed using Cutadapt (-a and -u, respectively) (http://cutadapt.readthedocs.io/en/stable/index.html, V1.9). Clean reads were mapped to the reference genome (V2.0, http://camelinadb.ca) using HISAT2 (2.0.4) (22) with default parameters. Reads aligned to genes were counted with the R package Rsubread (V1.32.2), using default parameters and allowing multi-mapping reads (23), and the transcript abundance estimated as transcripts per kilobase million (TPM).

### Database and web platform construction

The website sits on an Ubuntu 18.04 operating system, the current long-term support release, using a PostgreSQL database instance for backend storage and the Apache webserver for displaying pages. It was built on top of that base using the Drupal content management system with the Tripal and Tripal Analysis Expression modules along with their dependencies (24–26). The data were loaded into the Chado and Drupal database schemas using importers constructed using Tripal, and custom PHP codes were written to provide the functionality seen on the site today. The software and versions currently in use are PostgreSQL (10.12), PHP (7.1), Apache (2.4.41), Tripal (3.2), Tripal Analysis Expression (3.0) and Drupal (7.69).

### *Camelina sativa* gene annotation

All the functional annotation of *C. sativa* genes analyzed here, except for TFs (see below), were based on homology with *Arabidopsis thaliana* obtained by performing reciprocal BLAST analyses on ‘all proteins against all’, and from literature (8). The characterization and annotation of TFs and co-regulatory proteins (CoRs) assigned to the two databases harboring TFs and co-regulators (CsTFDB and CsCoTFDB, respectively) was carried out based on the identification of proteins that contain domains distinctive of these groups of proteins, as previously described (20, 27).

### Gene regulation data collection and analysis

To identify potential TFs, we utilized already existing knowledge of known and identified TF protein domains from published literature sources, particularly AGRIS and GRASSIUS (grassius.org) (20, 27). The data obtained were used in conjunction with Pfam’s Hidden Markov Models (HMM) to perform a domain search using the HMMER(v3) software against the predicted Camelina proteins sequences (8). Hit scores were only retained if they were considered significant, where the threshold used was a gathering score greater than the reported HMM for domains that are found in the Pfam database.

For those protein domain families that had no representative HMM in the Pfam database, custom-built HMM’s were created using alignment sequences obtained from PlnTFDB and published data (28). They were then built into HMM’s using HMMER, a calculated cut-off score for hits was established using the method described (28).

Once potential TFs were identified, they were classified based on already established domains rules. The rules consist of which protein domain or domains are required for a TF to be part of a certain family. In some instances, it involves not having a specific domain or set of domains (forbidden domains) to be classified as part of the specified family. The co-regulators were classified based on rules previously established (29). A modified version of the iTAK Perl script (30) was utilized to assign the proteins to families based on hits obtained from the hmmscan application in the HMMER Program.

### Gene co-expression analyses

The co-expression analyses between pairs of genes was calculated using the log_2_ of the TPMs as input data and the weighted Pearson correlation coefficient (PCC) as a metric for co-expression using the R package wCorr (Version 1.9.1) (31), with an optimal threshold of 0.4 to weight samples similarities.

## Results and discussion

### Database structure

**Figure 1.**
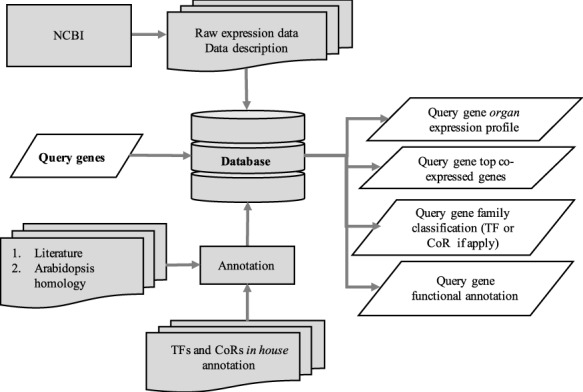
Schematic diagram outlining the architecture of CamRegBase 1.0.

**Figure 2.**
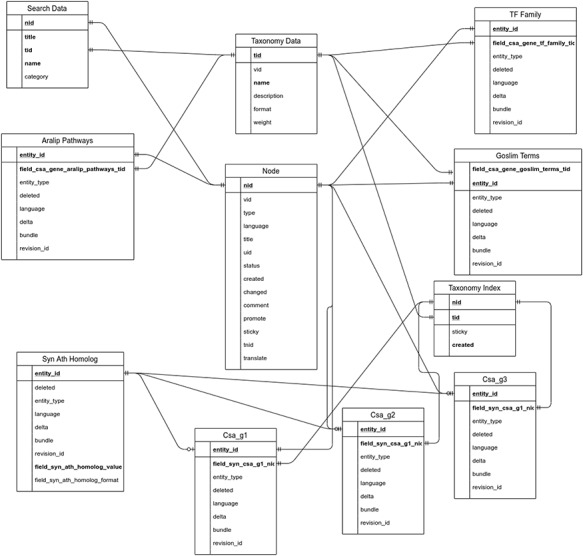
Entity relationship diagram of the main tables used in the backend of CamRegBase 1.0.

The utilization of the open source Tripal toolkit for the construction of the database web portal ensures that it can be expanded by the addition of compatible extension modules, and it ensures interoperability with a number of widely used biological knowledgebases (26). The overall database organization is schematized in Figure 1, with the search functionality of the site relying on the underlining database tables shown in the entity relationship diagram (Figure 2). All of the records in Drupal are stored in the ‘node’ table, which is queried in relation to the other tables on the search term provided by the end user. The lines shown in the diagram show how the tables are related when a search is run. For example, when a search is run using the Gene Search page, the ‘Search Data’ table is queried to return data matching the search term in the ‘title’, ‘name’ or ‘category’ fields. That table contains a consolidation of data from the ‘Node’ and ‘Taxonomy Data’ tables along with a ‘category’ value based on the presence of the record in any of the ‘Goslim Term’, ‘Aralip Pathway’ and/or ‘TF Family’ tables. The consolidation was done to improve the performance of the search function. Other searches query the tables directly. In the case of the Syntelogs search, the ‘Homolog’ is examined, and the data are returned along with related results from the ‘Csa_g1’, ‘Csa_g2’, ‘Csa_g3’ and ‘Taxonomy Data’ tables using the relationships shown in the entity relationship diagram.

### Expression database content

**Figure 3.**
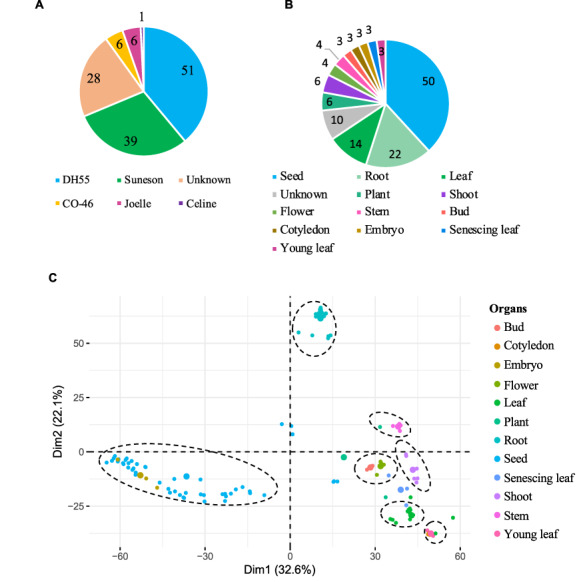
Gene expression data hosted on CamRegBase 1.0. Summary of expression data available on CamRegBase at the level of **(A)** Camelina varieties and **(B)** organ-specific samples. **(C)** PCA of the Camelina transcriptome using log_2_TPMs. Dotted ovals indicate major groups of samples identify by visual inspection of the PCA results.

The gene expression database was built based on 131 publicly available Camelina RNA-seq experiments (See ‘Materials and methods’). The data correspond to gene expression information from five different Camelina ‘varieties’, with DH55 and Suneson being the varieties with the largest number of samples (Figure 3A). Out of the 131 samples, 28 had no details in regard to the variety and thus were labeled as unknown and utilized solely for the co-expression analyses (See below). Data were classified on the basis of variety and further grouped on the basis of plant organs and seed development stages. In total, we analyzed data from 12 different ‘organs’, including whole plant pools (referred as ‘Plant’), and samples without ‘organ’ specification (defined as ‘Unknown’) (Figure 3B). Notably, seeds and roots represented the majority of samples available (38.8% and 16.8%, respectively) (Figure 3B). In terms of ‘seed developmental stages;, samples were analyzed that covered a range of 36 days post-anthesis (DPAs), with 14 different time points from 4 to 40 days post-anthesis (Supplementary Table 2). Overall, approximately four billion reads were analyzed, with an average of 29.9 million reads per sample and with 95.7% of the reads mapping to the genome (Supplementary Table 1).

To characterize the transcriptome at the sample level, the top 5% of genes with highest expression variation (TPMs) across all 131 samples were selected and a principal component analysis (PCA) was performed. The first two principal components explained 54.7% of the variation of the samples and allowed us to separate the 12 organs into 7 groups (Figure 3C). The ‘root’ and ‘seed’ samples grouped closest together and were the most distinct from the other samples. As expected, some samples aligned closely with others such as ‘embryo’ with ‘seed’ samples, ‘cotyledons’ with ‘young leaf’, and ‘buds’ with ‘flowers’ (dashed circles, Figure 3C). The observed separation suggests that, at least for the major groups, the data collected and presented here capture relevant biological information.

### Annotation of TFs

**Figure 4.**
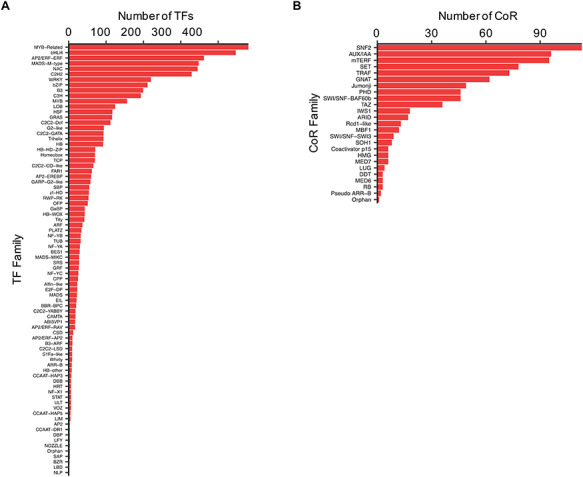
Distribution of the **(A)** TF and **(B)** CoR genes according to families, as currently present in CamRegBase 1.0.

TFs and CoRs play central roles in controlling gene expression, and they provide powerful tools to manipulate developmental or metabolic pathways for biotechnological purposes (11, 32). Thus, to characterize Camelina TFs and CoRs, advantage was taken of the current literature in this regard (21) and used to expande the previous collection using pipelines based on protein domain characterization and family classifications that worked well before in other plants (20, 27) (See ‘Materials and methods’). In total, 4619 TFs and 805 CoRs were identified of which 1075 TFs and 793 CoRs had not been previously reported (Supplemental Table 3). Our analysis, however, failed to identify 971 TFs previously reported based on homology (21). Currently, CamRegBase harbors information on 5590 TFs classified into 81 families, and 805 CoR, classified into 25 different families (Figure 4).

### Database functionalities

CamRegBase 1.0 consists of quick-buttons and tabs for navigation. The buttons are redundancies of the navigation tab. A unified search function within the ‘Gene Search’ tab was implemented, where a user may query Camelina genes by gene accession number, Arabidopsis GO Slim term or pathways from the Aralip database to find a gene of choice. Once a gene is selected, the resulting page provides gene information, a link to explore gene expression and a list of the top 50 co-regulated genes with their associated PCCs. When gene expression is explored, an expression analysis chart is displayed showing expression values across bio-sample numbers. Hovering over a data point will show the complete data information. Charts can also be downloaded in CSV format. Under the ‘Regulation’ tab a user may find a group of genes within a TF family, or they can go directly to the gene information page by searching with a Camelina gene accession number. Under the ‘Gene Expression’ tab, a user can go directly to gene expression information, or click on ‘Heat Map’ to view the selection of genes in a heat map, which can be sorted by gene name, annotation or blast description. A drop-down selection of the samples permits to visualize just a few or all the gene expression samples in the database. Alternatively, sample selection can also be done on the created heatmap by highlighting the desired samples; the heatmap will adjust accordingly. Finally, on the ‘Syntelogs’ tab, a user can query a Camelina or Arabidopsis gene accession number to see how they relate to one another.

### Future developments

CamRegBase 1.0 provides a first step toward building an inclusive platform for the integration of gene regulatory information in Camelina. All the data in CamRegBase are downloadable and readily available to the community. While co-expression information is presented for now in pre-assembled PCC tables, in future releases of CamRegBase this will be calculated and displayed in a dynamic fashion, allowing the immediate integration of new datasets, or the parsing of particular gene expression datasets to estimate co-expression values in particular tissues or conditions.

## Supplementary Material

baaa075_Supp
